# Toxic Algae Silence Physiological Responses to Multiple Climate Drivers in a Tropical Marine Food Chain

**DOI:** 10.3389/fphys.2019.00373

**Published:** 2019-04-04

**Authors:** Lucy M. Turner, Jonathan N. Havenhand, Christian Alsterberg, Andrew D. Turner, Girisha S. K, Ashwin Rai, M. N. Venugopal, Indrani Karunasagar, Anna Godhe

**Affiliations:** ^1^Department of Marine Sciences, University of Gothenburg, Göteborg, Sweden; ^2^Marine Biology and Ecology Research Centre, School of Biological and Marine Sciences, University of Plymouth, Plymouth, United Kingdom; ^3^Department of Biology, Lund University, Lund, Sweden; ^4^Centre for Environment, Fisheries and Aquaculture Science, Weymouth, United Kingdom; ^5^Department of Fishery Microbiology, College of Fisheries, Karnataka Veterinary Animal and Fisheries Sciences University, Mangalore, India; ^6^UNESCO-MIRCEN for Medical and Marine Biotechnology, Nitte University Centre for Science Education and Research, Nitte University, Mangalore, India

**Keywords:** *Meretrix*, Arabian Sea, multiple drivers, PSP, trophic/food chain, climate change, indirect effects, structural equation modeling

## Abstract

Research on the effects of climate change in the marine environment continues to accelerate, yet we know little about the effects of multiple climate drivers in more complex, ecologically relevant settings – especially in sub-tropical and tropical systems. In marine ecosystems, climate change (warming and freshening from land run-off) will increase water column stratification which is favorable for toxin producing dinoflagellates. This can increase the prevalence of toxic microalgal species, leading to bioaccumulation of toxins by filter feeders, such as bivalves, with resultant negative impacts on physiological performance. In this study we manipulated multiple climate drivers (warming, freshening, and acidification), and the availability of toxic microalgae, to determine their impact on the physiological health, and toxin load of the tropical filter-feeding clam, *Meretrix meretrix*. Using a structural equation modeling (SEM) approach, we found that exposure to projected marine climates resulted in direct negative effects on metabolic and immunological function and, that these effects were often more pronounced in clams exposed to multiple, rather than single climate drivers. Furthermore, our study showed that these physiological responses were modified by indirect effects mediated through the food chain. Specifically, we found that when bivalves were fed with a toxin-producing dinoflagellate (*Alexandrium minutum*) the physiological responses, and toxin load changed differently and in a non-predictable way compared to clams exposed to projected marine climates only. Specifically, oxygen consumption data revealed that these clams did not respond physiologically to climate warming or the combined effects of warming, freshening and acidification. Our results highlight the importance of quantifying both direct and, indirect food chain effects of climate drivers on a key tropical food species, and have important implications for shellfish production and food safety in tropical regions.

## Introduction

The ocean biome continues to be subjected to climate change (Hoegh-Guldberg and Bruno, 2010; Doney et al., 2012; Poloczanska et al., 2016). Key documented drivers are warming, acidification, and in coastal areas rainfall-driven freshening and oxygen depletion, all of which are projected to increase in severity (IPCC, 2014). We have a growing understanding of these as individual drivers on the physiology and ecology of selected marine organisms, particularly in those with temperate distributions (Wernberg et al., 2012). We are also beginning to characterize the varying synergistic, additive and antagonistic effects of these drivers in tandem, and in comparisons among marine species communities (Johnson et al., 2017; Legrand et al., 2017), populations (Calosi et al., 2017; Gibbons et al., 2017), life-history stages (Przeslawski et al., 2015; Marshall et al., 2016) and generations (Griffith and Gobler, 2017; Truebano et al., 2018). However, there remains a paucity of information on the effects of multiple climate change drivers in more complex, ecologically relevant settings, such as across trophic levels, and particularly in tropical marine organisms and ecosystems (Breitburg et al., 2015). Multi-trophic level studies are an important tool for furthering our understanding of the consequences these multiple drivers induce in marine ecosystems as they can explicitly provide insight into the effects of these on food web structure and ecosystem functioning and services (Estes et al., 2011; Goldenberg et al., 2017).

Tropical coastal marine ecosystems are projected to experience some of the most extreme effects of climatically-induced changes, including increased warming and freshening due to changes to monsoon-driven weather patterns (Bates et al., 2008) together with associated coastal eutrophication. Collectively these factors are known to cause structural changes to the oceanic microbial community in terms of both species abundance and composition (Boyce et al., 2010; Godhe et al., 2015), potentially leading to an increase in outbreaks of Harmful Algal Blooms (HABs) (Hallegraeff, 2010; Fu et al., 2012). Tropical coastal areas are also projected to be at risk from ocean acidification (OA) (Jennerjahn, 2012) with elevated *p*CO_2_ concentrations known to increase both the growth and toxicity of toxic phytoplankton communities (Kremp et al., 2012; Tatters et al., 2013; Errera et al., 2014; Hattenrath-Lehmann et al., 2015; Pang et al., 2017). Together these direct and indirect effects of marine climate change, mediated through changes in the marine microbial community, have been suggested to severely impact the tropical coastal marine food web (Turner et al., 2016).

Bivalves are key components of tropical coastal marine food webs (e.g., Lin et al., 2006; Lonsdale et al., 2009), and face both the direct, and the indirect, effects of marine climate change. The direct effects of warming, freshening and acidification on bivalve metabolic and immunobiological function are known to be significant and negative (Matozzo and Marin, 2011; Parker et al., 2013; Filgueira et al., 2016). As filter feeders, bivalves can accumulate algal toxins to extremely high levels, which in turn can have detrimental effects on their metabolism and immune system (Manfrin et al., 2012), as well as cascading effects on top consumers (Hallegraeff, 2010). Previous work in tropical southwest India demonstrated that bivalves (*Perna viridis*), exposed to simulated climate change and toxin-producing dinoflagellates showed significant shifts in metabolic and immunobiological function, which resulted in an increased toxin load (Turner et al., 2016). The bivalve *Meretrix meretrix* is an economically important subsistence species along the southwest Indian coast from which paralytic shellfish toxin (PST) contamination has previously been reported (Sharma et al., 2011). Prior studies on *M. meretrix* have demonstrated that elevated temperatures (Zhuang and Liu, 2006) and decreased salinity (Tang et al., 2005) can negatively influence metabolic function, causing an increase in metabolic rate (MO_2_) as well as changes to feeding physiology (ingestion rate and assimilation efficiency; Zhuang, 2006). Exposure to sub-lethal salinities can also have negative effects on behavior, including a decrease in sand clearance rates (Lui and Leung, 2004), whilst exposure to toxin-producing dinoflagellates has been documented to result in rapid acute lethality (Xu et al., 2018).

In the present study, we explored the effects of food source (non-toxic diatoms or toxin-producing dinoflagellates) combined with complex climate change scenarios on the links between the physiological health and toxin load (“toxicity”) in *M. meretrix*. In mesocosms we exposed clams to projected climate change conditions (warming and/or freshening and/or acidification) with or without the toxin producing dinoflagellate *Alexandrium minutum*. Total toxicity (PST concentration) was subsequently quantified and immunobiological as well as metabolic aspects of clam physiological function were examined. Using Structural Equation Modeling (SEM), we assessed the relative importance of direct and indirect effects of feed and climate drivers on the physiological pathways that ultimately affect clam physiological health and overall toxicity. We specifically hypothesized that: (i) warming, freshening and acidification would have detrimental effects on metabolic and immunobiological function in *M. meretrix* leading to changes in clam toxicity; and (ii) the indirect effects of climate drivers on physiological mechanisms and clam toxicity would be as strong as the direct effects.

## Materials and Methods

### Experimental Design and Setup

To investigate the combined effects of projected climate change and exposure to toxic phytoplankton on physiological function in *M. meretrix*, a nested experimental design was used. This incorporated two levels of seawater temperature, two salinities and two levels of *p*CO_2_ (Supplementary Figure 1). The two seawater temperature levels (28and 32°C) correspond to the mean monthly sea surface temperature (SST) at the collection site (Godhe et al., 2015) and to an increase of +4°C in line with projected warming trends for SST (IPCC, 2014). The two salinity levels (35 PSU and 31 PSU) correspond to the mean monthly sea surface salinity (SSS) at the collection site (Godhe et al., 2015) and to a decrease of 4 PSU as projected by future rainfall models (IPCC, 2014). The two levels of *p*CO_2_ correspond to current (∼400 μatm CO_2_/pH ∼8.1) and end of the century (∼1200 μatm CO_2_/pH ∼7.7) seawater projected values (Caldeira and Wickett, 2005; IPCC, 2014; see Supplementary Table 1 for details). Effects of *p*CO_2_ were only evaluated in conjunction with warming and freshening (e.g., 32°C + 31 PSU + 1200 μatm CO_2_) as we reasoned that this most accurately reflects combined future scenario projections for oceanic conditions (Caldeira and Wickett, 2005; IPCC, 2014; Boyd et al., 2018). For instance, an increase in oceanic *p*CO_2_ without warming (e.g., 28°C + 1200 μatm CO_2_) is unlikely.

Clams were exposed to either the non-toxic diatom *Thalassiosira weissflogii* or the PST producing dinoflagellate *Alexandrium minutum*. *Alexandrium minutum* is a widely distributed species responsible for paralytic shellfish poisoning events in coastal regions around the world (Anderson et al., 2012; Lewis et al., 2018), and has previously been recorded from the southwest Indian coast (Godhe et al., 2001). The concentration of *T. weissflogii* fed to the bivalves, 1000 cells/mL, corresponds to the amount used (based on carbon concentration) in similar experiments using bivalves (Lassus et al., 1994; Li et al., 2002). The amount of *A. minutum* used (100 cells/mL) provided the same concentration of carbon as 1000 cells/mL of *T. weissflogii* (Menden-Deuer and Lessard, 2000; Sun and Liu, 2003). The daily presence of feces and pseudofaces was used as an indicator of sufficient feed concentration (Riisgard et al., 2011).

After an initial habituation phase where clams were exposed to constant conditions for at least 5 days to remove any effects of differences in recent environmental history (see Supplementary Materials for details of animal collection and husbandry), clams were exposed for 14 days to either *T. weissflogii* or *A. minutum*, in one of the five climate change treatment combinations: “control” (28°C + 35 PSU + 400 μatm CO_2_), “warming” (32°C + 35 PSU + 400 μatm CO_2_), “freshening” (28°C + 31 PSU + 400 μatm CO_2_), “warming + freshening” (32°C + 31 PSU + 400 μatm CO_2_) or “warming + freshening + acidification” (32°C + 31 PSU + 1200 μatm CO_2_). For the groups exposed to warming, the water temperature in the aquaria (described above) was raised by 1°C per day until 32°C was achieved (day zero for all experimental exposures), by using aquarium immersion heaters. Similarly, for groups exposed to freshening, salinity was decreased by 1 PSU per day until 31 PSU was obtained, by adjustment with freshwater that had been filtered by reverse osmosis. *p*CO_2_ levels for the groups exposed to the high *p*CO_2_ treatment were controlled with NBS-calibrated pH-computers (Aqua Medic GmbH, Germany) with a precision of ± 0.01 pH units (∼12 μatm CO_2_). pH_NBS_ set-points equivalent to the desired *p*CO_2_ levels in the treatments were determined by measuring pH_NBS_ in seawater equilibrated for 12 h with custom mixed gases at 415 or 1240 μatm CO_2_ (Space Cryogases Pvt. Ltd., Mumbai, India). To control for possible fluctuations in alkalinity (which would change the pH set-points required to achieve an equivalent *p*CO_2_), calibrations were repeated every week during the experiment and the pH-computers adjusted accordingly. Carbonate system parameters were calculated using the CO2SYS software (Pierrot et al., 2006) within the CO2calc app (Robbins et al., 2010) employing constants from Mehrbach et al. (1973) refitted to the NBS pH scale by Dickson and Millero (1987) and the KSO_4_ dissociation constant from Dickson (1990), and also accounting for gas flux Wanninkhof (1992) and boron concentration (Lee et al., 2010). For high *p*CO_2_ treatments, *p*CO_2_ was increased, leading to a fall of ∼0.1 pH units per day until the experimental pH was reached (pH∼7.7).

Clams were divided haphazardly into two groups of 256, each assigned to either the *A. minutum* or non-toxic diatom control exposure regime described above. Each group was further sub-divided into four groups of 64 individuals and allocated to one of the five nested climate-change treatments, and then each of these were subdivided again into four groups of 16 individuals each allocated haphazardly to one of four replicate aquaria. Aquaria (volume = 20 L) were filled with aerated seawater that had previously been sand-filtered and ozonated. Temperature was maintained above ambient by placing individual aquaria into larger tanks, the water of which was heated using aquarium immersion heaters, thus avoiding any difficulties in temperature regulation in a small volume. Salinity and pH were decreased below ambient as before. Maximum stocking density was one clam per liter. Clams were fed daily as per their allocated microorganism exposure. All other experimental procedures were as described above.

At the end of the exposure period (14 days), the clams from each of the aquaria, were subdivided into three groups (see Supplementary Materials for full details of animal husbandry, all experimental procedures, and assay protocols). Individuals in group one were weighed and haemolymph was then taken from the posterior abductor muscle and added to an equal volume of Hepes-buffered physiological saline to assay for lysosomal membrane stability and thus immunobiological status (Neutral Red Retention Assay, see Supplementary Materials for methods). Clams were then opened, sexed, and the mantle tissue rapidly dissected and immediately frozen at −80°C for determination of ATP, ADP, AMP, glucose and glycogen levels, and thus cellular energy status. Gill tissue was also dected from these individuals, added to SEI buffer and frozen at −80°C for determination of gill function *via* Na^+^/K^+^-ATPase activity. Mantle and gill tissue samples were stored at −80°C until analysis. Group two clams were used for determination of metabolic rates (MO_2_) and thus oxidative metabolism. Clams from the third group were used for toxin (PST) quantification (see Supplementary Materials for detailed methods).

### General Linear Model (GLM) Analyses

**FIGURE 1:**
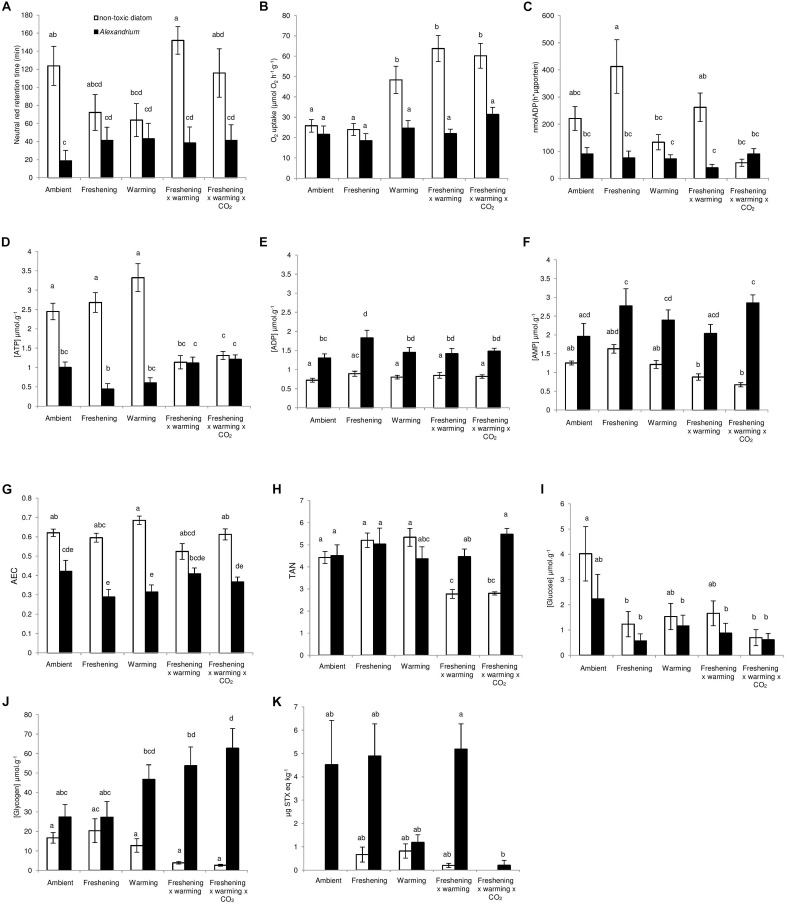
The interactive effects of *Alexandrium minutum* exposure and simulated climate change effects on some aspects of physiological function and PST concentration in *Meretrix meretrix*. The **(A)** immnobiological status (lysosomal membrane stability), **(B)** oxygen consumption **(C)** gill function, **(D)** mantle [ATP] (μmol g^−1^), **(E)** mantle [ADP] (μmol g^−1^), **(F)** mantle [AMP] (μmol g^−1^), **(G)** adenylate energy charge (AEC), **(H)** total adenylate nucleotides (TAN), **(I)** mantle [glucose] (μmol g^−1^), **(J)** mantle [glycogen] (μmol g^−1^), and **(K)** PST concentration (μg STX eq kg^−1^) of *M. meretrix* following 14 day’s exposure to the non-toxin producing diatom *Thalassiosira weissflogii* or the toxin producing dinoflagellate *A. minutum* under differing climate change conditions. Within each graph, different letters indicate a significant effect of *A. minutum* exposure for the simulated climate change scenario (*P* < 0.05), but see also Tables 1, 2. Data are means ± SEM. Samples sizes (*n* = 16) but see Supplementary Table 2.

Linear mixed effects models were used to investigate the effects of feed exposure and climate-change drivers on the mean physiological traits investigated, with “tank” as a random factor and individual body mass as covariate (Figure 1). Tests of normality and homogeneity of variances (Levene’s test, *P* > 0.05) indicated that some subsets of data did not fully meet the assumptions of standard parametric tests, even after transformation. However as GLM is generally robust against violations of normality (Underwood, 1997) all analyses were completed using this approach. In preliminary analyses, the terms “tank” and “body mass” were found to not have a significant effect on the parameters investigated (*P* ≥ 0.215), and were therefore removed. Significant differences among different treatments were further investigated *post hoc* using Tukey’s test. All analyses were conducted in SPSS v. 22.

### Structural Equation Modeling (SEM) Analyses

**FIGURE 2:**
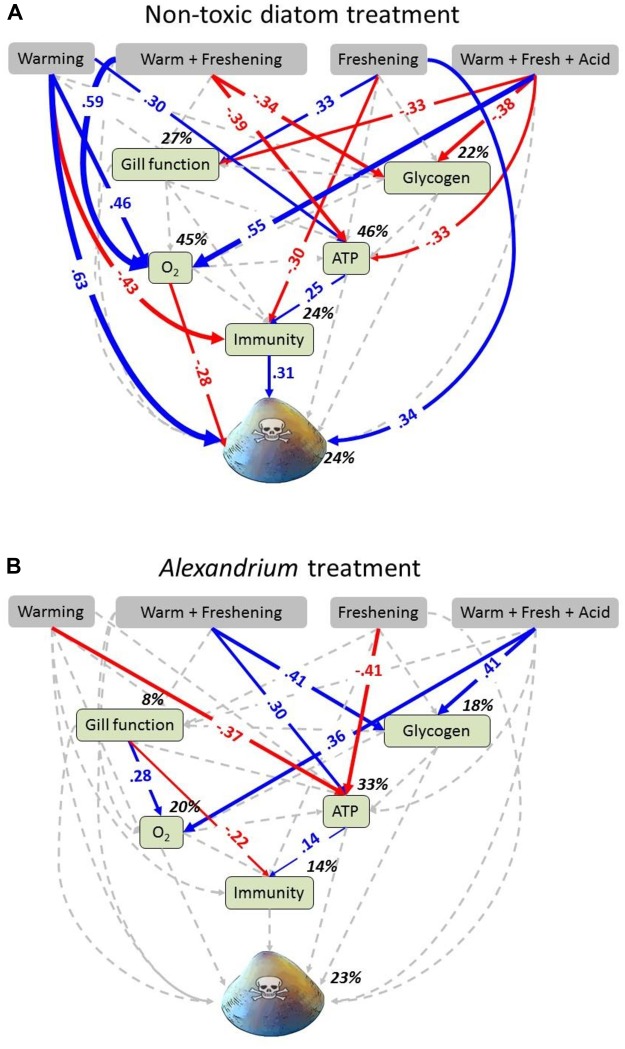
Path diagrams showing the interactive effects of exposure to food source and simulated climate change effects on toxin concentration in *Meretrix meretrix*. Path diagrams showing how experimental warming, freshening and the interaction between warming and freshening and warming, freshening and acidification affect the gill function, glycogen, oxygen consumption (O_2_), ATP concentration, immunity and toxin concentration. Path diagrams represent **(A)** clams fed with non-toxic diatoms (*Thalassiosira weissflogii*) and, **(B)** clams fed with *Alexandrium minutum*. Solid paths (blue and red) are (positively and negatively) statistically significant (*P* < 0.05) whereas the dashed gray lines are not. Note that a positive effect on “toxicity” results in an increased toxicity level within the clams, whereas a negative effect results in a decreased toxicity level. At each significant path the standardized coefficients are represented and interpreted as follows: If, for example, temperature goes up by 1 SD during *A. minutum* exposure, the ATP concentration of clams goes down by 0.37 SD. Percentages indicate the variance explained by the model. Samples sizes (*n* = 16) but see Supplementary Table 2.

We used SEM to separate and estimate the relative importance of direct and indirect effects of simulated climate change conditions from that of exposure to different food sources on the physiological pathways that affect the health of the clams, and their overall toxicity. Data were separated into two groups based on exposure to either the non-toxic diatom or *Alexandrium* and analyzed with a multigroup SEM (Grace, 2003, 2006). In the multigroup SEM, we assessed the effects of four binary predictor variables – (i) warming (0/1), (ii) freshening (0/1), (iii) warming + freshening (0/1), and (iv) warming + freshening + acidification (0/1) – and modeled their direct and/or indirect effects on six continuous response variables: (i) gill function; (ii) glycogen concentration; (iii) oxygen consumption; (iv) ATP concentration; (v) immunity (as lysosomal membrane stability measured as neutral red retention); and (vi) toxicity. Direct effects arise when variable A causes variable B to change (e.g., warming → O_2_ consumption), whereas indirect effects are the sum of all possible variables affecting variable B (e.g., the indirect effect of warming on O_2_ consumption is the sum of the path between warming → gill function, gill function → O_2_ consumption, warming → glycogen, glycogen → O_2_ consumption) (see also Figure 2 and Supplementary Tables 4, 5). First, we analyzed the data in each group to ensure that the basic structure of the model was consistent with the data. Data were analyzed by comparing models with the observed covariance matrix, using maximum likelihood and Chi-square as goodness of fit measures. Data were considered significantly different from the model when *P* < 0.05. Since data from the individual groups fit the model well (*P* ≥ 0.683), we deemed it legitimate to perform a multigroup SEM analysis. All variables in the model were initially constrained to vary equally across all groups. Standardized residual covariances, which display the difference between sample covariance and implied covariance, were then examined to locate variable inequalities between groups. Any inequalities that differed between the two groups by >2 in absolute values were relaxed (or allowed to vary freely across groups), and the analysis was run again. This stepwise procedure was performed until the model Chi-square no longer changed. Intercepts of the regression equations were also investigated to test differences for each endogenous variable between the two groups (Supplementary Table 4). Significance levels for individual paths between variables were set at α = 0.05. Structural equation models were run in AMOS (v. 20).

## Results

### Survival Rates

Survival rates were greater than 76% in most treatment groups. The exceptions to this were for those clams fed the non-toxic diatom and exposed to the triple-driver treatment (32.81%) and clams fed *Alexandrium* and exposed to warming (42.19%) (Supplementary Table 3).

### General Linear Model (GLM) Analyses

There were no significant effects of climate change drivers on immune function (measured as lysosomal membrane stability *via* neutral red retention) in clams fed the non-toxic diatom. However, exposure to *Alexandrium* had a significant negative effect on immune function that interacted significantly with the simulated climate change treatments with negative effects seen in clams exposed to *Alexandrium* under ambient as well as single and both multi-driver treatments (Figure 1A and Table 1). There was a significant increase in oxygen consumption with exposure to warming – either singly, or in combination with other drivers – but only in clams fed the non-toxic diatom. For clams fed with *Alexandrium*, there was no substantive effect of any of the simulated climate change treatments (Figure 1B and Tables 1, 2). Exposure to *Alexandrium* had a predominantly significant negative effect on Na^+^/K^+^-ATPase activity. However, a significant interaction between the effects of simulated climate change conditions and *Alexandrium* exposure meant that there was no significant difference in Na^+^/K^+^-ATPase activity in the triple-driver treatment between non-toxic diatom fed and *Alexandrium* fed clams (Figure 1C and Tables 1, 2). For the remaining metrics of energy status, simulated climate change had broadly consistent significant and negative effects in clams fed the non-toxic diatom (Figure 1D–H and Tables 1, 2) with the most negative effects often seen in the two- and three-driver treatments (Figure 1D,F,H). Here ATP concentration and total adenylate nucleotides (TAN) were reduced when clams were exposed to freshening + warming and freshening + warming + acidification while there was no change in ADP or AMP concentration or AEC (Figure 1D–H). By comparison, in general for clams fed with *Alexandrium* the ATP concentration and AEC were reduced when compared to clams fed the non-toxic diatom. For clams fed *Alexandrium* the magnitude of the response to any of the simulated climate change treatments did not vary significantly. Measured concentrations of glucose and glycogen showed very different patterns of response: simulated climate change conditions (all treatments) reduced concentrations of glucose regardless of the presence of toxic microalgae; while glycogen concentrations showed a similar pattern, this was not significant, and they did so only in the absence of *Alexandrium* – in the presence of *Alexandrium* glycogen concentrations increased under the triple-driver climate change treatment (Figure 1I,J and Tables 1, 2).

**Table 1:** GLM results of the effects of simulated climate change and exposure to *Alexandrium minutum* and their interaction on clam toxicity, immunobiological status, oxidative metabolism, gill function and cellular energy status in the Asiatic hard clam *Meretrix meretrix.*

Parameter	Climate change	*A. minutum* exposure	Climate change + Microorganism exposure
**Toxicity**			
PST (μg STX eq kg^−1^)	*F*_4_ = 1.13, *P* = 0.349	***F*_1_ = 8.61, *P* = 0.004**	*F*_4_ = 1.62, *P* = 0.176
**Immunobiological Status**			
Neutral red retention	*F*_4_ = 1.830, *P* = 0.126	***F*_1_ = 36.396, *P* = 0.000**	***F*_4_ = 2.837, *P* = 0.027**
**Oxidative Metabolism**			
O_2_ uptake (μmol O_2_ h^−1^ g^−1^) *Gill function*	***F*_4_ = 14.438, *P* = 0.000**	***F*_1_ = 65.394, *P* = 0.000**	***F*_4_ = 4.871, *P* = 0.001**
Na^+^/K^+^-ATPase	***F*_4_ = 4.456, *P* = 0.002**	***F*_1_ = 26.059, *P* = 0.000**	***F*_4_ = 5.243, *P* = 0.001**
**Cellular Energy Status**			
ATP (μmol g^−1^)	***F*_4_ = 4.972, *P* = 0.001**	***F*_1_ = 89.768, *P* = 0.000**	***F*_4_ = 9.739, *P* = 0.000**
ADP (μmol g^−1^)	***F*_4_ = 3.000, *P* = 0.021**	***F*_1_ = 114.088, *P* = 0.000**	*F*_4_ = 1.043, *P* = 0.387
AMP (μmol g^−1^)	***F*_4_ = 3.035, *P* = 0.019**	***F*_1_ = 82.766, *P* = 0.000**	***F*_4_ = 2.954, *P* = 0.022**
AEC	*F*_4_ = 1.037, *P* = 0.391	***F*_1_ = 88.205, *P* = 0.000**	***F*_4_ = 4.970, *P* = 0.001**
TAN	***F*_4_ = 5.732, *P* = 0.000**	***F*_1_ = 7.078, *P* = 0.009**	***F*_4_ = 7.741, *P* = 0.000**
Glucose (μmol g^−1^)	***F*_4_ = 5.205, *P* = 0.001**	*F*_1_ = 3.652, *P* = 0.058	*F*_4_ = 0.577, *P* = 0.680
Glycogen (μmol g^−1^)	*F*_4_ = 0.893, *P* = 0.470	***F*_1_ = 61.136, *P* = 0.000**	***F*_4_ = 6.263, *P* = 0.000**

*Detailed test results for statistically significant effects of simulated climate change are shown in Table 2. F-values are given with degrees of freedom in subscript. Significant values (P < 0.05) are highlighted in bold. AEC, adenylate energy change. TAN, total adenylate nucleotides.*

**Table 2:** Parameter estimates and Tukey *post hoc* test results of the effects of simulated climate change (warming, freshening, warming + freshening, warming + freshening + acidification) on oxidative metabolism, gill function and cellular energy status in the Asiatic hard clam *Meretrix meretrix*.

Parameter	Warming	Freshening	Warming + Freshening	Warming + Freshening + Acidification
**Oxidative Metabolism**				
O_2_ uptake (μmol O_2_ h-^1^ g-^1^)	**40.133, *T* = 3.99, *P* = 0.001**	21.234, *T* = −0.35, *P* = 0.997	**42.864, *T* = 4.58, *P* = 0.000**	**45.793, *T* = 5.25, *P* = 0.000**
**Gill Function**				
Na^+^/K^+^-ATPase	102.722, *T* = −1.22, *P* = 0.742	244.131, *T* = 2.04, *P* = 0.251	150.637, *T* = −0.10, *P* = 1.000	73.841, *T* = −1.80, *P* = 0.379
**Cellular Energy Status**				
ATP (μmol g^−1^)	1.259, *T* = 0.03, *P* = 1.000	1.110, *T* = −1.77, *P* = 0.394	**1.016, *T*** = −**3.05, *P* = 0.023**	1.103, *T* = −1.94, *P* = 0.302
ADP (μmol g^−1^)	1.127, *T* = 1.16, *P* = 0.775	**1.360, *T* = 3.41, *P* = 0.008**	1.133, *T* = 1.22, *P* = 0.743	1.152, *T* = 1.38, *P* = 0.642
AMP (μmol g^−1^)	1.804, *T* = 0.91, *P* = 0.892	2.202, *T* = 2.66, *P* = 0.066	1.460, *T* = −0.68, *P* = 0.959	1.763, *T* = 0.70, *P* = 0.956
TAN	2.170, *T* = 0.92, *P* = 0.888	2.232, *T* = 1.61, *P* = 0.491	**1.843, *T*** = −**2.92, *P* = 0.033**	2.000, *T* = −1.04, *P* = 0.838
Glucose (μmol g^−1^)	**1.350, *T*** = −**2.99, *P* = 0.026**	**0.902, *T*** = **3.60, *P* = 0.004**	**1.269, *T*** = −**3.13, *P* = 0.018**	**0.656, *T*** = −**4.05, *P* = 0.001**

*Results shown only for parameters with statistically significant effects (Table 1). T-values are given. Significant values (P < 0.05) are highlighted in bold. TAN, total adenylate nucleotides.*

Exposure of clams to *Alexandrium* increased clam PST concentration (Figure 1K and Table 1). However, the magnitude of the response varied among simulated climate change treatments (Figure 1K), with the biggest increases in PST seen in clams exposed to ambient conditions, freshening, or freshening + warming. Warming (alone) and the combination of freshening + warming + acidification both reduced PST levels substantially (Figure 1K).

### Structural Equation Modeling (SEM) Analyses

Individual and multigroup SEM models were statistically similar to the observed data (Table 3). SEM analyses revealed strong direct and indirect effects of climate change drivers on clam metabolic and immunobiological function, although the physiological response differed with exposure to either the non-toxic diatom or *Alexandrium* (Figure 2). The exception to this was that oxygen consumption increased under the triple-driver treatment under both feeding regimes.

**Table 3:** SEM statistics.

Models	χ^2^	df	*P*
**Individual Group Models**			
Control group, diatoms	0.167	1	0.683
*Alexandrium* treatment	0.003	1	0.959
Multigroup model	3.243	6	0.778

*Chi-square (χ^2^) likelihood tests for the individual and multigroup SEM models. When P ≥ 0.05, fitted models are not significantly different from observed data.*

For the clams fed the non-toxic diatom, SEM showed strong direct positive effects of warming (0.63) and freshening (0.34) on toxicity (Figure 2A). Direct and indirect effects of climate change (all treatments) on immunity also caused an increase in toxicity (0.31) (Figure 2A). By comparison, effects of warming as well as the double- and triple-driver climate change treatment on oxygen consumption caused a decrease in toxicity (−0.28) (Figure 2A). However, in those clams fed *Alexandrium*, the SEM did not demonstrate any direct or indirect effects of climate change on overall toxicity (Figure 2B). Comparisons of intercepts for each measured variable between the two groups (non-toxic diatoms vs. *Alexandrium*) (Supplementary Table 4) also showed that the model changed significantly when gill function, ATP, toxicity as well as glycogen were set as equal across groups. Thus, exposure to *Alexandrium* directly affected these variables (as also shown by the GLM analyses; Table 1 and Figure 1). Standardized total, direct and indirect effects for the groups “non-toxic diatom” and “*Alexandrium*” are given in Supplementary Tables 5a,b.

## Discussion

We found that exposure of bivalves to projected marine climates resulted in direct and indirect negative effects on physiological function, and that these effects were often more pronounced in clams exposed to multiple, rather than single climate drivers. In addition, our study demonstrates that physiological responses of bivalves to projected climate change conditions can be modified by interactions within the food chain, specifically by what bivalves eat. Our experiments were relatively short (14 days), and longer-term investigations are now required to further elucidate the effects of climate change on marine systems. Nonetheless, our results demonstrate the importance of quantifying both direct and, indirect food chain effects of climate drivers in order to widen our understanding of marine climate change on species and ecosystems.

In our experiments, clams fed the non-toxic diatom and exposed to warming upregulated their metabolic activity (demonstrated by an increase in oxygen consumption and ATP production). This increase in ATP production also drove a concomitant increase in TAN. This increase in metabolism (as MO_2_) was also more pronounced in clams that were exposed to warming in combination with freshening and/or acidification and is a typical response of bivalves, including *M. meretrix* to increased temperatures (Zhuang and Liu, 2006). This is due to thermodynamic changes in physiological processes, as well as multi-driver climate change conditions (Matoo et al., 2013). This was also reflected in the high mortality rates for clams exposed to the triple-driver treatment. The SEM showed that glycogen concentrations simultaneously decreased in clams exposed to the multi-driver climate scenarios which suggests that this increase in metabolism is being fuelled by glycogenolysis and ultimately glycolysis. The results of the SEM showed that exposure to freshening led to an increase in gill function and decrease in immune function, signifying the importance of maintaining homeostatic ion-osmotic mechanisms rather than immune function under reduced salinity conditions (Gajbhiye and Khandeparker, 2017). However, when instead clams were exposed to the triple-driver treatment, the SEM showed that gill function decreased. There were also significant decreases in ATP, TAN, and glucose concentrations. This suggests that under these conditions the upregulation in oxygen consumption was not sufficient to maintain the energetically costly ion-osmotic mechanism.

When bivalves were fed with the toxin-producing dinoflagellate, *Alexandrium*, exposure to multiple climate change treatments significantly affected clam physiology, with various effects between individual stressors, including the mechanisms underpinning toxin uptake. Clams fed *Alexandrium* responded very differently to those fed the non-toxic diatom. For example, glycogen levels were consistently higher in all treatments where clams were fed the toxin-producing dinoflagellate. This is in agreement with previous work on okadaic acid, another marine toxin produced by several phytoplankton species and responsible for diarrhetic shellfish poisoning in humans which has revealed that this toxin is able to induce alterations in several metabolic pathways including glycogen synthesis (Valdiglesias et al., 2012). There were also significant decreases in oxygen consumption (as MO_2_) and ATP production in *Alexandrium* fed clams compared to those fed the non-toxic diatom suggesting that exposure to toxin producing dinoflagellates caused a downregulation of the clam’s metabolic machinery. By comparison, TAN increased reflecting the higher proportion of ADP and AMP concentrations compared to ATP in these clams and at the same time there was an associated decrease in AEC further signifying the decrease in energy status of these clams when challenged with exposure to *Alexandrium*. Exposure of bivalves to phycotoxins has been previously recorded to cause changes to metabolic processes, including those involved in detoxification pathways (Manfrin et al., 2012). In our experiment these decreases in measured metabolic parameters were also more pronounced in clams exposed to multi-stressor climate change conditions. This suggests that clams fed with *Alexandrium* were not able to respond to environmental challenges that in normal conditions would increase their metabolic rate, such as an increase in temperature. This could indicate that these clams had to shut down some functions totally when they were facing these environmental challenges which may ultimately lead to time limited survival rates if they are not able to adjust rapidly.

However, in terms of toxicity, in non-toxic diatom fed clams, there were negligible levels of PST present, reflecting the fact that despite 5 days depuration prior to use in our experiment these clams retained some of the background level of PST from the wild. The fact that these levels of PST were negligible probably explains why the SEM and GLM analyses gave different results, e.g., significant vs. no significant differences in toxicity of these clams with climate change exposure. However, in GLM analysis of clams fed with a toxin-producing dinoflagellate, warming reduced the toxin load and this was further reduced when exposed to warming in combination with freshening and acidification, although this result was not significant. Our results contradict previous suggestions that toxicity of bivalves exposed to toxin-producing phytoplankton species may increase when also exposed to climate change drivers (e.g., freshening, warming, and/or elevated *p*CO_2_): first of all because of the direct effects of these climate drivers on phytoplankton growth and toxicity levels (Hallegraeff, 2010), and secondly because of the unpredictable indirect effects of climate drivers on bivalve physiology (Turner et al., 2016) which in turn may also affect feeding rates (Sara et al., 2008) and therefore toxin uptake. Thus, our findings add to the growing number of studies showing that our understanding of marine climate change will further be improved by experiments that investigate the effects of multiple climate change drivers in complex, ecologically relevant settings that include multiple trophic levels (Alava et al., 2017; Boyd et al., 2018).

Our findings from the SEM analyses, are in accordance with previous studies that demonstrate reduced toxicity levels of bivalves exposed to PST-producing phytoplankton in combination with multiple climate change drivers (specifically, warming, or freshening, warming and acidification) (Farrell et al., 2015; Braga et al., 2018). These studies point to the well-characterized impacts of increased temperature, and to some extent decreased pH on increasing metabolic function (and indirectly, clearance rates) as a mechanism for increasing detoxification rates. Increased temperature has a direct thermodynamic effect on metabolism (Seebacher et al., 2010), whereas decreased pH, can instead impact metabolic processes reliant on haemolymph pH such as gaseous exchange for respiration and excretion (Gazeau et al., 2013). However, this explanation is not reflected in our data for the whole organism or for cellular metabolism, which remained broadly constant under climate change conditions, and were likewise revealed to not have any significant effects on toxicity. An alternative mechanism for the reduced toxicity levels under climate change conditions may have been mediated *via* the effects of reduced pH on upregulating digestive enzyme activities and antioxidant production needed to maintain nutrient absorption. These processes may, in turn have a bearing on detoxification rates. This theory has recently started to gain traction (for a full discussion, see Braga et al., 2018). Several studies have recorded an increase in the activity of digestive enzymes and antioxidants such as glutathione in bivalves under high *p*CO_2_ conditions (e.g., Hu et al., 2015). Notably, glutathione in particular has been shown to have a fundamental role in the specific biotransformation and elimination of PST analogs (Costa et al., 2012). However, previous work has also shown that toxin uptake rates were reduced under warming and/or acidification in mussels (*Mytilus galloprovincialis*) fed a toxin-producing dinoflagellate, although the mechanism for this (e.g., increased metabolic rate, reduced clearance rate and/or action of digestive enzymes) was not characterized. Nevertheless, in the same study, detoxification rates when fed a non-toxic diatom were faster in mussels maintained under ambient conditions (Braga et al., 2018). The results from our experiment also contradict the results from one of our previous studies, which demonstrated that despite an upregulation in metabolic and immunobiological machinery, during *Alexandrium* exposure combined with climate change conditions (freshening and/or warming), toxicity levels of the green mussel, *P. viridis* increased (Turner et al., 2016). It can also not be overlooked that *M. meretrix* in the current study accumulated very low amounts of PST (on average < 6 μg STX eq kg^−1^) compared to recognized food safety limits (e.g., in Europe of 800 μg STX eq kg^−1^). Previous work has suggested that clams including *Meretrix* spp. may have a degree of resistance to PST uptake (Lu and Hwang, 2002), but other studies have reported very high PST levels of >3240 μg STX eq kg^−1^, e.g., from *Meretrix casta* during a HAB outbreak in southwest India (Karunasagar et al., 1984). However, the results presented in this study, together with results of previous studies suggest that further work is required to fully understand the mechanistic effects of multiple climate change drivers on uptake and detoxification rates by commercially important bivalves.

Our work has important implications for seafood producers and consumers. Firstly, the negative effects on clam physiological health of the climate change drivers reported here will likely have implications for shellfish producers (both farmers and fishermen) in terms of future yield (Cheung et al., 2010; Barange et al., 2014). Our results show a reduction in bivalve toxicity when also exposed to multiple climate change drivers (specifically, warming, or freshening, warming and acidification) but not under freshening, or freshening and warming scenarios. However, due to the fact that climate change drivers are known to negatively affect detoxification rates the implications for subsistence and commercial producers and consumers in terms of seafood safety are less clear. Our findings also highlight the need for further work on understanding the effects of future projected multi-driver climate change on tropical marine systems, an area that to date remains unexplored. This study is to our knowledge the first fully experimental investigation of multiple climate change drivers on a tropical food chain to be conducted in South Asia. As tropical coastal marine ecosystems, including those bordering the Indian Ocean are projected to experience some of the most extreme effects of climatic induced changes (Bates et al., 2008), more effort is urgently required to understand the long-term consequences for these areas where huge numbers of people depend on seafood as a protein and/or economic resource.

In a broader context, our work addresses the importance of including multiple climate drivers and trophic levels in experiments (Breitburg et al., 2015; Hurd et al., 2018). For example, our finding that physiological responses to multiple climate drivers changed fundamentally when bivalves were exposed simultaneously to a toxin-producing algal feed illustrate the complexity of responses and the need for environmentally relevant experiments. Our observation that the addition of more drivers (two or three rather than one) generally resulted in decreased organism performance is also of broader relevance to understanding the impacts of multiple climate drivers on marine organisms. Despite the fact that our experiment was relatively short (14 days) our results show the negative impact of multiple drivers on the physiology underpinning clam homeostasis. As with all experiments, our results are specific to the organisms and drivers we studied and therefore more such studies, especially over longer time scales, are required to build an empirical base against which relevant theory can be tested.

## Data Availability

The datasets generated for this study are available on request to the corresponding author.

## Ethics Statement

This study involved invertebrate species only which are outside the remit of Swedish ethical licensing requirements.

## Author Contributions

LT, JH, AG, and IK conceived the study. LT, JH, AR, GK, MV, and AG carried out the experiments. LT was responsible for the biochemical determinations. AT carried out the clam toxin analyses. LT and CA carried out the statistical analyses. LT, JH, CA, and AG led the writing. All authors commented on the final version of the manuscript.

## Conflict of Interest Statement

The authors declare that the research was conducted in the absence of any commercial or financial relationships that could be construed as a potential conflict of interest.
