# Supplementary material for: Toxic Algae Silence Physiological Responses to Multiple Climate Drivers in a Tropical Marine Food Chain
Source: Front Physiol. 2019 Apr 4;10:373. doi: 10.3389/fphys.2019.00373 (PMC6458267; doi:10.3389/fphys.2019.00373)
Supplement: Supplementary file 1 [file Data_Sheet_1.docx]

**Supplementary materials for ‘Toxic algae silences physiological responses to multiple climate drivers in a tropical marine food chain’**

Lucy M. Turner, Jonathan N. Havenhand, Christian Alsterberg, Andrew D. Turner, Girisha S.K., Ashwin Rai, M.N. Venugopal, Indrani Karunasagar, Anna Godhe

**Supplementary Tables**

**Supplementary Table 1** | Average pH, salinity and temperature (mean ± s.d.) during the 14 d experiment to examine the exposure to different simulated climate change conditions and/or *Alexandrium minutum* on *Meretrix meretrix*.

| Microorganism exposure | Climate change | pH_NBS_ | Salinity (PSU) | Temperature (°C) |
| --- | --- | --- | --- | --- |
| Non-toxic diatom | Ambient | 8.07 ± 0.02 | 34 ± 2 | 27.3 ± 0.5 |
|  | Freshening | 8.10 ± 0.03 | 31 ± 1 | 27.1 ± 0.5 |
|  | Warming | 8.04 ± 0.05 | 35 ± 1 | 31.1 ± 1.0 |
|  | Freshening + warming  Freshening + warming + acidification | 8.07 ± 0.04  7.74 ± 0.03 | 30 ± 0  30 ± 1 | 31.2 ± 1.0  30.8 ± 1.0 |
| *Alexandrium* | Ambient | 8.07 ± 0.04 | 35 ± 0 | 29.3 ± 0.8 |
|  | Freshening | 8.06 ± 0.05 | 31 ± 0 | 29.1 ± 0.8 |
|  | Warming | 8.07 ± 0.04 | 35 ± 0 | 32.5 ± 1.0 |
|  | Freshening + warming | 8.04 ± 0.05 | 31 ± 0 | 33.2 ± 0.8 |
|  | Freshening + warming + acidification | 7.65 ± 0.16 | 31 ± 0 | 33.5 ± 1.8 |

**Supplementary Table 2 |** Sample sizes (n) for each analysis conducted on *Meretrix meretrix* after 14 d exposure to different simulated climate change conditions and/or *Alexandrium minutum*.

|  |  | Toxicity | | | Immunobiological status | Oxidative metabolism | Gill function | Cellular energy status |
| --- | --- | --- | --- | --- | --- | --- | --- | --- |
| Microorganism exposure | Climate change | Toxin-pathogen load | PST |  | Neutral red retention | O_2_ uptake | Na^+^/K^+^-ATPase | ATP, ADP, AMP, AEC, TAN, Glucose, Glycogen |
| Non-toxic diatom | Ambient | 12 | 8 |  | 16 | 15 | 14 | 16 |
|  | Freshening | 15 | 7 |  | 16 | 16 | 15 | 16 |
|  | Warming | 16 | 8 |  | 16 | 16 | 15 | 16 |
|  | Freshening + warming | 14 | 6 |  | 16 | 15 | 16 | 16 |
|  | Freshening + warming + acidfication | 5 | 1 |  | 11 | 15 | 12 | 13 |
| *Alexandrium* | Ambient | 11 | 10 |  | 16 | 14 | 14 | 16 |
|  | Freshening | 16 | 17 |  | 16 | 16 | 16 | 12 |
|  | Warming | 16 | 16 |  | 16 | 16 | 16 | 16 |
|  | Freshening + warming | 11 | 11 |  | 16 | 16 | 10 | 16 |
|  | Freshening + warming + acidfication | 10 | 10 |  | 16 | 16 | 14 | 16 |

Where sample size n<16 this was the result of clam mortality during 14 day exposure to different simulated climate change conditions and/or *Alexandrium minutum* exposure (**Supplementary Table 3**). For toxicity determination, samples were pooled (see Supplementary Methods for details.)

**Supplementary Table 3 |** Survival rates for *Meretrix meretrix* after 14 d exposure to different simulated climate change conditions and/or *Alexandrium minutum*.

| Microorganism exposure | Climate change | % Survival |
| --- | --- | --- |
| Non-toxic diatom | Ambient | 93.75 |
|  | Freshening | 85.94 |
|  | Warming | 92.19 |
|  | Freshening + warming  Freshening + warming + acidification | 79.69  32.81 |
| *Alexandrium* | Ambient | 78.13 |
|  | Freshening | 85.94 |
|  | Warming | 42.19 |
|  | Freshening + warming | 76.56 |
|  | Freshening + warming + acidification | 79.69 |

**Supplementary Table 4 |** Investigation of model intercepts between groups with chi squares (*χ2*).

| Variable | Intercept different across groups | Intercept equal across groups | Difference in model *χ2* |
| --- | --- | --- | --- |
| Gill function | 3.243 | 11.860 | 8.617 |
| Glycogen | 3.243 | 4.771 | 1.528 |
| O_2_ consumption | 3.243 | 3.234 | 0 |
| ATP | 3.243 | 31.974 | 28.731 |
| NRRT | 3.243 | 3.898 | 0.655 |
| Toxicity | 3.243 | 9.546 | 6.303 |

First, all intercepts were allowed to differ across groups. Second, each variable was investigated by setting the intercept as equal across groups, which changed the model *χ2*. The difference in model *χ2* indicates whether the intercept is significantly different across groups.

**Supplementary Table 5a |** Standardised total, direct and indirect effects for the control group.

|  | Salinity | Temp | T×S | TxSxA | Glyco. | Gill f. | O_2_ c. | ATP | NRRT |
| --- | --- | --- | --- | --- | --- | --- | --- | --- | --- |
| Standardized total effects |  |  |  |  |  |  |  |  |  |
| Glycogen | 0.099 | -0.106 | -0.344 | -0.379 | 0.000 | 0.000 | 0.000 | 0.000 | 0.000 |
| Gill function | 0.318 | -0.157 | 0.066 | -0.271 | -0.146 | 0.000 | 0.000 | 0.000 | 0.000 |
| O_2_ c. | -0.028 | 0.454 | 0.589 | 0.533 | 0.031 | 0.026 | 0.000 | 0.000 | 0.000 |
| ATP | 0.076 | 0.286 | -0.432 | -0.356 | 0.019 | -0.029 | -0.052 | 0.000 | 0.000 |
| NRRT | -0.256 | -0.298 | 0.139 | 0.060 | -0.004 | 0.094 | 0.147 | 0.256 | 0.000 |
| Toxicity | 0.254 | 0.338 | 0.169 | 0.000 | -0.014 | 0.038 | -0.218 | -0.194 | 0.315 |
| Standardized direct effects |  |  |  |  |  |  |  |  |  |
| Glycogen | 0.099 | -0.106 | -0.344 | -0.379 | 0.000 | 0.000 | 0.000 | 0.000 | 0.000 |
| Gill function | 0.333 | -0.173 | 0.016 | -0.326 | -0.146 | 0.000 | 0.000 | 0.000 | 0.000 |
| O_2_ c. | -0.039 | 0.462 | 0.599 | 0.553 | 0.034 | 0.026 | 0.000 | 0.000 | 0.000 |
| ATP | 0.081 | 0.307 | -0.394 | -0.330 | 0.017 | -0.028 | -0.052 | 0.000 | 0.000 |
| NRRT | -0.302 | -0.428 | 0.149 | 0.093 | 0.000 | 0.097 | 0.160 | 0.256 | 0.000 |
| Toxicity | 0.345 | 0.638 | 0.170 | 0.034 | 0.002 | 0.007 | -0.278 | -0.275 | 0.315 |
| Standardized indirect effects |  |  |  |  |  |  |  |  |  |
| Glycogen | 0.000 | 0.000 | 0.000 | 0.000 | 0.000 | 0.000 | 0.000 | 0.000 | 0.000 |
| Gill function | -0.014 | 0.015 | 0.050 | 0.055 | 0.000 | 0.000 | 0.000 | 0.000 | 0.000 |
| O_2_ c. | 0.012 | -0.008 | -0.010 | -0.020 | -0.004 | 0.000 | 0.000 | 0.000 | 0.000 |
| ATP | -0.006 | -0.021 | -0.038 | -0.026 | 0.002 | -0.001 | 0.000 | 0.000 | 0.000 |
| NRRT | 0.046 | 0.131 | -0.010 | -0.032 | -0.004 | -0.003 | -0.013 | 0.000 | 0.000 |
| Toxicity | -0.091 | -0.300 | -0.001 | -0.034 | -0.016 | 0.030 | 0.061 | 0.081 | 0.000 |

Total effects are the sum of all direct and indirect effects. Direct effects are the direct effects of one variable on another variable, and indirect effects are sum of all products affecting one variable (e.g., the indirect effect of Temp on O_2_ consumption is the product of the path between Temp → Gill function, Gill function → O_2_ consumption, Temp → Glycogen, Glycogen → O_2_ consumption). T×S (Temperature × Salinity), T× S×A (Temperature × Salinity × Acidification), Glyco. (Glycogen), Gill f. (gill function), O_2_ c. (oxygen consumption), NRRT (immunity).

**Supplementary Table 5b |** Standardised total, direct and indirect effects for the *Alexandrium* group.

|  | Salinity | Temp | T×S | TxSxA | Glyco. | Gill f. | O_2_ c. | ATP | NRRT |
| --- | --- | --- | --- | --- | --- | --- | --- | --- | --- |
| Standardized total effects |  |  |  |  |  |  |  |  |  |
| Glycogen | -0.022 | 0.222 | 0.303 | 0.406 | 0.000 | 0.000 | 0.000 | 0.000 | 0.000 |
| Gill function | -0.059 | -0.079 | -0.276 | 0.025 | 0.134 | 0.000 | 0.000 | 0.000 | 0.000 |
| O_2_ c. | -0.042 | 0.150 | 0.065 | 0.359 | 0.016 | 0.282 | 0.000 | 0.000 | 0.000 |
| ATP | -0.412 | -0.360 | 0.062 | 0.139 | 0.088 | -0.037 | -0.059 | 0.000 | 0.000 |
| NRRT | 0.143 | 0.155 | 0.126 | 0.143 | -0.020 | -0.284 | -0.209 | 0.140 | 0.000 |
| Toxicity | 0.060 | -0.268 | 0.113 | -0.315 | -0.189 | 0.101 | 0.028 | -0.062 | 0.009 |
| Standardized direct effects |  |  |  |  |  |  |  |  |  |
| Glycogen | -0.022 | 0.222 | 0.303 | 0.406 | 0.000 | 0.000 | 0.000 | 0.000 | 0.000 |
| Gill function | -0.056 | -0.109 | -0.316 | -0.030 | 0.134 | 0.000 | 0.000 | 0.000 | 0.000 |
| O_2_ c. | -0.026 | 0.177 | 0.150 | 0.361 | -0.022 | 0.282 | 0.000 | 0.000 | 0.000 |
| ATP | -0.414 | -0.373 | 0.033 | 0.124 | 0.092 | -0.020 | -0.059 | 0.000 | 0.000 |
| NRRT | 0.180 | 0.218 | 0.068 | 0.201 | 0.001 | -0.222 | -0.201 | 0.140 | 0.000 |
| Toxicity | 0.035 | -0.245 | 0.199 | -0.240 | -0.196 | 0.094 | 0.026 | -0.063 | 0.009 |
| Standardized indirect effects |  |  |  |  |  |  |  |  |  |
| Glycogen | 0.000 | 0.000 | 0.000 | 0.000 | 0.000 | 0.000 | 0.000 | 0.000 | 0.000 |
| Gill function | -0.003 | 0.030 | 0.041 | 0.054 | 0.000 | 0.000 | 0.000 | 0.000 | 0.000 |
| O_2_ c. | -0.016 | -0.027 | -0.084 | -0.002 | 0.038 | 0.000 | 0.000 | 0.000 | 0.000 |
| ATP | 0.002 | 0.013 | 0.030 | 0.016 | -0.004 | -0.017 | 0.000 | 0.000 | 0.000 |
| NRRT | -0.036 | -0.063 | 0.057 | -0.058 | -0.021 | -0.062 | -0.008 | 0.000 | 0.000 |
| Toxicity | 0.025 | -0.023 | -0.086 | -0.075 | 0.007 | 0.007 | 0.002 | 0.001 | 0.000 |

Total effects are the sum of all direct and indirect effects. Direct effects are the direct effects of one variable on another variable, and indirect effects are sum of all products affecting one variable (e.g., the indirect effect of Temp on O_2_ consumption is the product of the path between Temp → Gill function, Gill function → O_2_ consumption, Temp → Glycogen, Glycogen → O_2_ consumption). T×S (Temperature × Salinity), T× S×A (Temperature × Salinity × Acidification), Glyco. (Glycogen), Gill f. (gill function), O_2_ c. (oxygen consumption), NRRT (immunity).

**Additional details for Methods**

**Animal collection and husbandry**

Clams (*Meretrix meretrix,* Linnaeus, 1758) (14.65 ± 0.18 g wet mass) were collected from Bengre, Mangalore, India (12° 52’ 55” N, 74° 49’ 27” E). Individuals were immediately transferred to the experimental aquarium facility <60 min after collection. Upon arrival clams were exposed to constant conditions for at least five days to remove any effects of differences in recent environmental history. This was achieved by placing the clams in a number of aquaria (volume= 200 L) filled with aerated seawater *T* (°C) = 28, *S* (PSU) = 35 that had previously been sand filtered and ozonated to remove the microbial community. Stocking density was at a maximum of one clam per two litres. Clams were exposed to a 12h:12h L:D cycle and fed the non-toxin producing diatom *Thalassiosira weissflogii* (Instant Algae TW1200, Reed Mariculture Inc, Campbell, CA, USA) once daily at a concentration of 1000 cells/mL. Every 1-2 d faeces and pseudofaeces were removed from the aquaria and half of the water was exchanged to remove metabolic waste products. Temperature and salinity were measured daily and corrected if needed.

**Culture of microorganisms**

A strain of *A. minutum* (CCMP113) was obtained from the Gothenburg University Culture Collection, Gothenburg, Sweden. The algae were cultured on site in f/2 medium (Guillard, 1975) based on 0.5 µm filtered natural seawater adjusted to 26 PSU at 25 °C on a 12h:12h L:D cycle and irradiance of 50 µmol photons m^-2^ s^-1^.

**Lysosome membrane stability**

Lysosome membrane stability was evaluated in clam haemocytes using the Neutral Red Retention Assay (Martinez-Gomez et al., 2008; Buratti et al., 2013). In healthy clams lysosomes will retain the acidophilic vital dye neutral red. Cellular toxicity will result in the integrity of the membrane being compromised and subsequent leakage of the dye (Moore et al., 2008). Briefly glass microscope slides were coated with 5 % poly-L-lysine and left to dry in a humid chamber for 30 min. Next, for each sample, 40 µL of the haemolymph-saline mixture (as previously described) was added in the same position where the poly-L-lysine was added. Slides were placed into a dark humid chamber for 30 min to allow cells to attach. After 30 min the slides were placed onto their side to allow excess solution to run off. Next 40 µL of a freshly prepared neutral red working solution (5 µL/mL saline from a stock solution of 20 mg neutral red dye dissolved in 1 mL DMSO) was added to the area containing the attached cells and a cover slip was applied. Slides were incubated for 15 min and then inspected under a microscope (40X) at increasing intervals (15-60 min) for a total of 180 min to determine at what point in time the dye that had been readily taken up into the lysosomal compartment of the cells, was lost to the remainder of the cytosol. The test for an individual sample was terminated when the lysosomal dye loss was evident in 50% (numerically assessed per field of view) of the haemocytes, and the time recorded. Data are expressed as Neutral Red Retention Time (Martinez-Gomez et al., 2008).

**Determination of gill Na^+^/K^+^-ATPase activity**

Gill samples were defrosted on ice, pulse centrifuged and the SEI buffer decanted, following which samples were transferred to 2 mL microcentrifuge tubes. Next, 1.2 mL SEI deoxycholate buffer was added (0.1 % Na deoxycholic acid in SEI buffer) and the samples were homogenised with a bead beater (MM300, Retsch, Düsseldorf, Germany) using glass beads (Sigma-Aldrich, Poole, UK). The supernatant was analysed for Na^+^/K^+^-ATPase activity (McCormick, 1993) in a microplate format using a Varioskan Flash plate reader (Thermo Scientific, Waltham, MA USA) equipped with proprietary software (SkanIt Software 2.4.3).

**Determination of metabolite concentration**

Mantle levels of ATP, ADP, AMP, glucose and glycogen were determined using standard assays. Beforehand mantle extracts were prepared (Bergmeyer, 1985a,b). Briefly, mantle samples were removed from storage at -80 °C and a known mass was transferred to chilled 2 mL microcentrifuge tubes. Next, the samples were homogenised with four parts 0.9 mol L^-1^ HClO_4_ using a bead beater and glass beads, following which the homogenate was centrifuged for 10 min at 20,000 *g* at 4 ºC. The homogenate was then transferred to a second 2 mL microcentrifuge tube. To neutralise the effects of the acid, four parts K_2_CO_3_, 3.75 mol L^-1^ for five parts of HClO_4_ was added and the tubes were then centrifuged for 10 min at 20,000 *g* at 4 ºC. The resulting supernatant was removed and used for the following assays.

Concentrations of ATP, ADP, AMP, glucose and glycogen were determined spectrophotometrically in the mantle samples. All assays were undertaken in a microplate format using a Varioskan Flash plate reader. Mantle ATP, ADP and AMP concentrations were determined using NADH linked assays (Bergmeyer, 1985b). Glucose and glycogen concentrations were measured using the hexokinase method (Bergmeyer, 1985a). Glycogen was measured as glucose (hexokinase method) before and after treatment with glycoamylase. The total adenylate content (TAN) for each individual was calculated by summing the concentrations of the three adenylates measured (ATP, ADP and AMP). The adenylate energy charge (AEC) was calculated using the equation: AEC = (ATP+½ADP)/(ATP+ADP+AMP).

**Determination of standard MO_2_**

Standard MO_2_ was determined using closed respirometry. Respirometry chambers (volume = 500 mL) were filled with aerated, clean (sand filtered, ozonated and GF/F filtered) seawater at the respective experimental temperature and salinity and a magnetic flea added. A platform (50 x 50 x 15 mm) above the magnetic flea prevented contact between the clam and the magnetic flea. A clam was added to each chamber, which was then sealed while submerged to prevent air bubbles. The chambers were loosely covered with aluminium foil to ensure that disturbance to the clam was minimised. Chambers were then placed onto magnetic stirrers (Remi Laboratory Instruments, Mumbai, India) to ensure adequate mixing of seawater and to prevent stratification of oxygen within the chamber. Before measurements began clams were allowed to settle in the chambers for 1 h, which is the minimum time required for establishing resting MO_2_. Planar optode spots (diameter 0.5 cm; PreSens Precision Sensing GmbH, Regensburg, Germany) were glued to the inside of each chamber. Oxygen levels in the chambers were measured every 5 min for a period of 1 h using a Fibox 4 oxygen meter (PreSens Precision Sensing GmbH) and PreSens Datamanager software (PreSens Precision Sensing GmbH). All the equipment was located inside rooms where the appropriate temperature level was maintained. For each chamber the decline in *p*O_2_ was linear over the measurement period and was never allowed to fall to hypoxic levels. Background respiration was taken into account by running blanks, and the average value across a number of blanks was subtracted from the original MO_2_ value. MO_2_ was expressed as µmol O_2_ h^-1.^g^-1^. Upon completion of measurements of MO_2_, clams were removed from the chambers, gently blotted dry and weighed. Clam volume was also obtained by displacement.

**Toxin extraction and analysis**

Analysis of PST was conducted using liquid chromatography with tandem mass spectrometry (LC-MS/MS). Instrument solvents used for mobile phase preparation were of LC-MS-grade (Fisher Optima, ThermoFisher, UK) and all chemicals were LC-MS reagent grade where possible. All other reagents were HPLC grade. Clams were first shucked and homogenised. Tissue homogenates were subjected to a single dispersive extraction using 1% acetic acid (Boundy et al., 2015), with post-centrifuge supernatants desalted using pre-conditioned Supelclean ENVI-Carb 250mg/3 mL solid phase extraction (SPE) cartridges. 100 µL of SPE eluant from each sample was diluted in 300 µL acetonitrile prior to LC-MS/MS analysis. Chromatographic separation of PST was performed using Hydrophilic Interaction Liquid Chromatography (HILIC). A Waters Acquity UPLC I-Class was used with a 1.7 µm, 2.1x150 mm Waters Acquity BEH Amide UPLC column in conjunction with a Waters VanGuard BEH Amide guard cartridge. The columns were held at +60^°^C, with samples held in the autosampler at +4^o^C. MS/MS detection was conducted using a Waters Xevo TQ-S tandem quadrupole mass spectrometer. Selected reaction monitoring (SRM) was conducted to enable the detection and quantitation of 19 different saxitoxins analogues (Turner et al., 2015). Quantitation of toxins in clam tissue extracts was performed through external calibration using toxin standard calibrants prepared at six different concentration levels from certified reference toxin solutions (Institute of Biotoxin Metrology, National Research Council Canada (NRCC), Halifax, Nova Scotia, Canada). Toxin concentrations were calculated in µmoles/kg shellfish tissue and converted to saxitoxin dihydrochloride equivalents per kg shellfish tissue (STX di-HCl eq / kg) using toxicity equivalence factors (TEFs) taken from EFSA recommendations (European Food Safety Authority, 2009).

**Supplementary Figure 1 |** Experimental design and setup.

This setup was replicated for each microorganism exposure regime, e.g. non-toxic diatom *Thalassiosira weissflogii* or the PST producing dinoflagellate *Alexandrium* *minutum*.

**B**

**D**

**E**

**D**

**A**

**E**

**B**

**A**

**E**

**E**

**D**

**A**

**A**

**C**

**C**

**B**

**D**

**C**

**C**

**B**

A = 28 °C + 35 PSU + 400 µatm CO_2_ (control)

B = 28 °C + 31 PSU + 400 µatm CO_2_ (freshening)

C = 32 °C + 35 PSU + 400 µatm CO_2_ (warming)

D = 32 °C + 31 PSU + 400 µatm CO_2_ (warming + freshening)

E = 32 °C + 31 PSU + 1200 µatm CO_2_ (warming + freshening + acidification)

Each tank contained 16 clams which were randomly assigned to four groups at the end of the exposure time:

Group one (four clams): i) Haemolymph was taken for lysosomal membrane stability

ii) Mantle tissue was taken for ATP, ADP, AMP, glucose and glycogen determination.

iii) Gill tissue was taken for determination of gill Na^+^/K^+^-ATPase activity.

Group two (four clams): Determination of metabolic rates (MO_2_).

Group three (four clams): Toxin quantification.

Group four (four clams): Not analysed.

**References**

Bergmeyer, H. (1985) *Methods of enzymatic analysis. Vol 6, Metabolites 1: Carbohydrates*. Weinheim, Germany: VCH Verlagsgesellschaft.

Bergmeyer, H. (1985) *Methods of enzymatic analysis. Vol 7, Metabolites 2: Tri and dicarboxylic acids, purine, pyrimidines, inorganic compounds*. Weinheim, Germany: VCH Verlagsgesellschaft.

Boundy, M. J., Selwood, A. I., Harwood, D. T., McNabb, P. S., and Turner, A. D. (2015) Development of a sensitive and selective liquid chromatography-mass spectrometry method for high throughput analysis of paralytic shellfish toxins using graphitic carbon SPE. *J. Chromatogr. A.* 1387, 1-12.

Buratti, S., Franzellitti, S., Poletti, R., Ceredi, A., Montanari, G., Capuzzo, A., and Fabbri, E. (2013) Bioaccumulation of algal toxins and changes in physiological parameters in Mediterranean clams from the North Adriatic Sea (Italy). *Environ. Toxicol.* 28, 451-470.

European Food Safety Authority. (2009) Scientific opinion of the panel on contaminants in the food chain on a request from the European Commission on marine biotoxins in shellfish - saxitoxin group. *EFSA Journal.* 1019,1-76.

Guillard, R. R. L. (1975) “Culture of phytoplankton for feeding marine invertebrates,” in Culture of Marine Invertebrate Animals, eds. W. L. Smith and M. H. Chanley (New York: Plenum Press), 26-60.

Martinez-Gomez, C., Benedicto, J., Campillo, J. A., and Moore, M. (2008) Application and evaluation of the neutral red retention (NRR) assay for lysosomal stability in clam populations along the Iberian Mediterranean coast. *J. Environ. Monitor.* 10, 490-499.

McCormick, S. D. (1993) Methods for nonlethal gill biopsy and measurement of Na^+^, K^+^-ATPase activity. *Can. J. Fish. Aquat. Sci.* 50, 656-658.

Moore, M. N., Kohler, A., Lowe, D., and Viarengo, A. (2008) “Lysosomes and autophagy in aquatic animals,” in Autophagy: Lower Eukaryotes and Non-Mammalian Systems, Pt A Methods in Enzymology, ed. D. J. Klionsky (San Diego: Elsevier Academic Press Inc.), 581-620.

Turner, A. D., McNabb, P. S., Harwood, D. T., Selwood, A. I., and Boundy, M. J. (2015) Single-laboratory validation of a multitoxin ultra-performance LC-hydrophilic interaction LC-MS/MS method for quantitation of paralytic shellfish toxins in bivalve shellfish. *J. AOAC Int.* 98, 609-621.
